# Fairness informs social decision making in infancy

**DOI:** 10.1371/journal.pone.0192848

**Published:** 2018-02-14

**Authors:** Kelsey Lucca, Jacqueline Pospisil, Jessica A. Sommerville

**Affiliations:** Department of Psychology, University of Washington, Seattle, Washington, United States of America; Fordham University, UNITED STATES

## Abstract

The ability to reason about fairness plays a defining role in the development of morality. Thus, researchers have long been interested in understanding when and how a sensitivity to fairness first develops. Here, we examined infants’ ability to use fairness information in selecting social partners. Using a novel experimental paradigm that combined pre-recorded stimuli with an active behavioral measure, we tested whether infants preferred to socially engage with an individual they had previously seen behave fairly or unfairly. After viewing an individual distribute goods to third parties either equally (i.e., 3:3 distribution) or unequally (i.e., 5:1 distribution), both 13- and 17-month-old infants selectively chose to engage in a social interaction with (i.e., take a toy from) an individual who distributed resources equally. The use of a novel paradigm to assess infants’ fairness preferences demonstrates that infants’ previously established fairness preferences extend across different, more demanding paradigms, and may therefore be more enduring in nature. Together, these findings provide new insights into the nature of infants’ fairness representations, and fill in key gaps in the developmental timeline of infants’ ability to use fairness information in their consideration of potential social partners. In sum, these findings build on previous research by demonstrating that infants not only hold an expectation that resources should be distributed fairly, they also preferentially interact with those who have previously done so. The early-emerging ability to both reason about and use fairness information may play an influential role in the development of complex prosocial behaviors related to morality more broadly.

## Introduction

One of the foundational features of human morality is the tendency to act fairly and value fairness. Across cultures, there is a universal assumption that individuals should behave fairly [1–3]. Although there are different theoretical accounts of what behaviors are considered fair, one of the most prominent models is that of distributive justice: holding all else constant, resources should be distributed to recipients equally [4]. The fair distribution of resources activates the brain’s reward circuitry [5], and when resources are not distributed fairly, individuals react negatively [6]. Adults are so averse to inequity that they will often incur costs to avoid unequal distributions [7]. Thus, not surprisingly, adults prefer individuals who have a history of distributing resources fairly [8].

Given the critical importance of fairness to adults, researchers have long been interested in pinpointing when and how a sensitivity to fairness develops. Researchers have established that the tendency to distribute resources fairly develops early: 18-month-olds will spontaneously divide resources equally [9], 3-year-olds will verbally correct situations in which an equality norm is violated [10], and by 6 years of age, children will dispose of resources rather distribute them unequally [3,11]. While these findings provide important information regarding when children begin to act fairly, if a sensitivity to fairness is a developmental building block of more sophisticated moral behaviors, an implicit awareness of and preference for fairness and fair actors likely develops before children have the abilities to execute fair behaviors or verbally articulate fairness concerns.

There are two ways researchers have probed infants’ early, pre-verbal sensitivity to fairness: by testing whether infants (1) expect individuals to act fairly and (2) prefer individuals who act fairly. Although these abilities may appear similar, at least in principle, they could involve very different processes [12]. Infants’ fairness expectations demonstrate that they have a pre-conceived notion about how resources typically are distributed. This expectation may be a result of prior exposure to equal resource distributions: infants may have learned the statistical regularities with which equal resource distributions occur. Alternatively, infants may view unequal distributions as a transgression of social norms. Regardless, infants can have an expectation about how events typically, or should, proceed without necessarily attaching valence to these events. In contrast, infants’ preference for social partners inherently involves infants’ assignment of valence to different individuals: infants must rank order different individuals in order to choose one as a social partner over the other.

Although infants’ expectations and social preferences may involve distinct processes, researchers often study these two abilities interchangeably, using different methodologies, in order to make claims about the developmental trajectory of infants’ fairness representations (or other socio-moral representations) more broadly. However, whether these processes do in fact emerge simultaneously, or whether they follow distinct development trajectories, is an empirical question. Determining whether infants’ fairness expectations, and infants’ use of fairness information to select of social partners, follow similar or different developmental trajectories is critical for understanding the origins of infants’ sensitivity to fairness, and the nature of infants’ socio-moral knowledge and behavior more generally.

### Fairness expectations

To investigate infants’ early fairness expectations, researchers have utilized violation-of-expectancy (VOE) paradigms which test whether fairness transgressions draw enhanced visual attention from infants. For example, upon viewing individuals distribute resources fairly (2:2 distribution of resources) and unfairly (3:1 distribution of resources) to third party recipients, infants between 12 and 15 months of age look longer at unfair distributions [12,13]. Infants’ increased looking towards unequal distributions suggests that it violates their expectations about how third-party fairness interactions typically play out. In a series of follow-up studies, researchers have investigated the developmental origins of infants’ fairness expectations. Findings from Ziv and Sommerville (2016) revealed that at 6- and 9-month-olds do not hold fairness expectations. A study by Meristo and colleagues (2016) found that just one month later, at 10 months of age, infants hold fairness expectations—suggesting that infants’ fairness expectations first begin to emerge around 10-months of age [14].

At 10 months of age, as infants fairness expectations are first emerging, there is evidence to suggest that these expectations are already quite sophisticated: infants hold an affiliative expectation that unfair individuals should not, or typically are not, approached by third parties [15,16]. As infants enter their second year of life, their fairness expectations become even more complex. Infants expect resource distributions to align with social dominance structures [17] and only expect resources to be distributed equally when both receiving parties have equally earned those resources [18]. Infants also hold expectations about how fair and unfair individuals should be treated: fair individuals should be praised, whereas unfair individuals should be admonished [19].

### Social selections on the basis of fairness

While tests of infants’ fairness expectations require minimal effort from infants (i.e., they must either look or not look at different stimuli), tests of infants’ preference for fair individuals are much more demanding. In social preference tasks, infants must first rank their relative evaluations of two or more individuals, which requires them to keep track of which individuals exhibited fair or unfair behaviors. Then, infants must integrate their decision with a motor behavior, in most cases reaching, in order to establish their preference. Despite the complexity of this type of task, a recent study by Burns and Sommerville (2014) found that infants as young as 15 months are capable of using an individual’s prior history of acting fairly to guide their selection of social partners. In this study, infants preferred to engage in a social interaction with (i.e., take toys from) an individual who previously distributed resources fairly, as opposed to unfairly. Importantly, this does not appear to be true of younger infants: 10-month-olds do not demonstrate a preference for fair over unfair individuals [20].

### The development of fairness preferences

Taken together, the studies outlined above provide converging evidence that infants expect resources to be distributed fairly, and prefer to affiliate with individuals who have previously done so. While the literature on the development of infants’ fairness expectations is relatively extensive, much less is known about the developmental trajectory of infants’ use of fairness information to guide social preferences. Specifically, it remains unknown whether infants’ preference for fair individuals follows the same trajectory as the established developmental trajectory of infants’ fairness expectations. If the underlying processes of these two measures of infants’ fairness sensitivity develop simultaneously, fairness preferences should be present during infancy once fairness expectations have emerged. Alternatively, it may be that infants’ preferences for fair individuals are reflective of a more mature component of fairness sensitivity because infants must weigh their relative evaluations of others while at the same time making an active decision about how to behave. If this is the case, then fairness preferences should only develop after fairness expectations are fully established, and may be present at the start of the second year of life, or not within infancy at all.

In addition to the open questions surrounding when infants’ preferences for fair individuals first emerges, questions also remain surrounding the continuity of this preference across development. Infants show a preference for fair individuals starting at 15 months, and while it is intuitive to assume that this preference would also be present later in development, previous research has consistently shown that developmental abilities are often not continuous [21]. Infants may prioritize fairness when selecting a social partner early in development, but then weigh other traits more heavily when selecting a social partner later in development. The time between 15 and 18 months is a particularly informative time to test hypotheses surrounding developmental differences in infants’ social preferences because infants’ social abilities are undergoing a major transition: the time spent engaging in joint attention with others doubles [22], and communication skills are growing at an exponential rate [23]. These changes may influence how infants weigh different characteristics when they select social partners. Thus, to understand infants’ preference for fair individuals as it relates to their overall sensitivity to fairness, it is critical for researchers to fully establish the developmental unfolding of infants’ social preference for fair individuals.

### Assessing infants’ fairness preferences across paradigms

In addition to assessing infants’ fairness preferences across development, it is important to assess fairness preferences across paradigms. Assessing infants’ fairness preferences across different paradigms has the potential to provide important insights into the nature of these preferences. Traditionally, research on infants’ fairness preferences has used manual choice measures, in which infants indicate their preference through reaching [24–26]. This type of assessment makes the act of selection easy for infants, as infants begin reaching as early as 3 months [27]. In the current study, we made the act of establishing a social preference more difficult for infants. Specifically, we had infants select a social partner using a more recently developed and difficult to produce behavior: crawling or walking combined with reaching. Rather than placing two toys directly in front of the infant, as in prior work, we positioned them in out-of-reach boxes such that infants had to first leave the comfort of their caregivers’ lap, then crawl or walk towards the boxes, and finally combine these behaviors with reaching into a box to search for a toy.

By requiring infants to establish their social preference using more effortful and complex behaviors than previously used measures, the current study makes several, inter-related contributions to the literature on infants’ fairness preferences. Most broadly, by testing whether infants’ fairness preferences replicate across different measurements we aimed to provide new insights into the nature of infants’ underlying fairness representations. Indeed, different behavioral measures that attempt to assess the same underlying construct often produce different results [21]. These dissociations provide important information about the nature of the underlying representation that is being assessed [28]. For instance, studies using looking-time paradigms to assess infants’ object permanence find evidence that infants can mentally represent hidden objects [29,30], whereas studies on older children using reaching paradigms fail to replicate this finding [31,32]. Rather than interpreting these discrepancies as evidence that object permanence is discontinuous across development, researchers have argued that infants have a weak representation of object permanence, meaning, their object permanence is graded, or only partially-developed, when measured early in life [33]. If infants’ fairness preferences are also graded, and not fully adult-like in nature, we would predict that paradigms with fewer task demands (e.g., reaching measures) would find evidence that infants prefer fair individuals, whereas paradigms that place additional demands on infants, such as the one used in the current study, would find no evidence for fairness preferences. Alternatively, if fairness preferences (based on the principle or norm of equality) are already robust and quite sophisticated during infancy, we would expect to find evidence of fairness preferences, regardless of how they are measured.

The paradigm used in the current study places three additional physical and cognitive demands on infants, compared to previously used reaching paradigms. First, it requires that infants integrate multiple different behaviors together. For infants to be successful in our task, they had walk or crawl and combine that behavior with reaching and search behaviors. Second, it calls for infants to produce a behavior that is less well-rehearsed than simply reaching: infants begin reaching as early as 3 months, whereas they only begin walking at around 12 months of age. Finally, the behaviors infants needed to produce in the current paradigm take more time to produce than reaching alone. Thus, any preferences that emerge in the current study is likely indicative of a more deliberate choice and enduring representation than those assessed in previously used reaching paradigms. Reaching paradigms that require infants to make a quick and easy-to-produce selection may only be tapping into a momentary and fleeting preference.

In using a novel paradigm to assess infants’ fairness preferences, we also aimed to provide important pragmatic insights into infants’ early social preferences. Testing infants’ preferences across paradigms allows us to understand the conditions under which infants’ fairness preferences can be replicated, a question that is particularly important in the current climate of concerns about both direct and conceptual replications. Unlike previous studies that have examined infants’ fairness preferences either by using pre-recorded stimuli or live actors [20,25], the current design exposed infants to fairness information using pre-recorded visual stimuli, but assessed infants’ social preferences with an active behavioral measure (adapted from [33]). Specifically, we simultaneously combined pre-recorded stimuli with a forced-choice task to create the illusion that infants had the choice to socially interact with a fair or unfair individual who appeared on a projector screen. This novel method allowed us to capture infants’ naturalistic social behavior while maintaining the experimental control that is afforded by using pre-recorded stimuli (e.g., the presentation of stimuli was held constant, we could confirm that infants were attentive to the display).

### The current study

In sum, the current study set out to fulfill two key objectives. First, we tested whether infants use fairness information in their selection of social partners at two previously unstudied developmental milestones, 13 and 17 months. In doing so, we aimed to provide new insights into whether different markers of fairness sensitivity (e.g., preference for fair individuals, fairness expectations) follow similar or different developmental trajectories. Second, we tested the efficacy of a novel experimental design in assessing infants’ preference for fair individuals, thereby evaluating the extent to which infants’ preference for fair individuals extends across more physically and cognitively demanding, ecologically-valid contexts. This allowed us to examine the nature and strength of infants’ fairness representations. Given that infants have developed fairness expectations by approximately 10 months of age, we predicted that across both ages, infants would selectively interact with individuals who behave fairly, compared to unfairly. Together, this work will provide new insights into infants’ ability to use fairness information in their consideration of potential social partners. This information is critical because infants’ selection of social partners has a direct influence on their early social and cognitive development. Who infants prefer as social partners shapes who they interact with, and the kind of information they are subsequently exposed to.

## Methods

### Participants

Fourteen 13-month-old infants (8 females; mean age = 13 months, 11 days; range = 12 months 21 days to 13 months 9 days) and sixteen 17-old infants (10 females; mean age = 17 months, 10 days; range = 16 months 27 days to 18 months 19 days) participated. Infants were typically developing and born at full-term. All participants were White, and parents identified their education level as having a college degree or higher (*n* = 25) or having some college (*n* = 7).

Participants were recruited from a database of parents who had volunteered to participate in experimental studies. Parental consent was obtained for each participant. Data from nineteen additional infants were excluded due to lack of response (*n* = 11), fussiness (*n* = 5), failing to reach an attention criterion of an average of 80% to all distribution events (*n* = 2) or technical errors (*n* = 1). The experiment took place in a research laboratory at a large university in the Pacific Northwest. After participation each infant received a toy as compensation.

### Procedure

**Fig 1:**
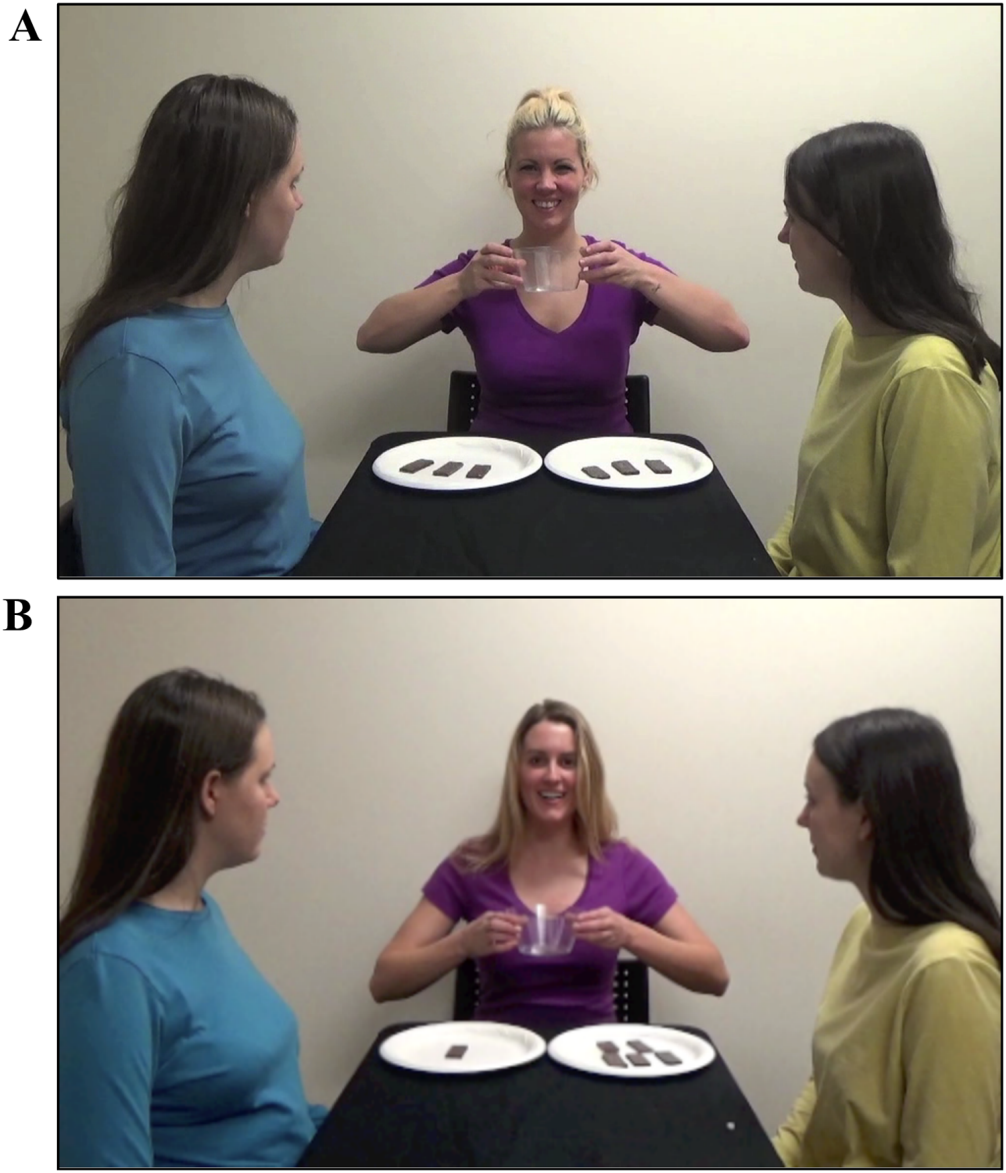
Distribution event stimuli. A still frame from the videos of a fair distribution event (A) and an unfair distribution event (B). All individuals appearing in all photographic figures are members of the research team and have consented to have their images used here.

**Fig 2:**
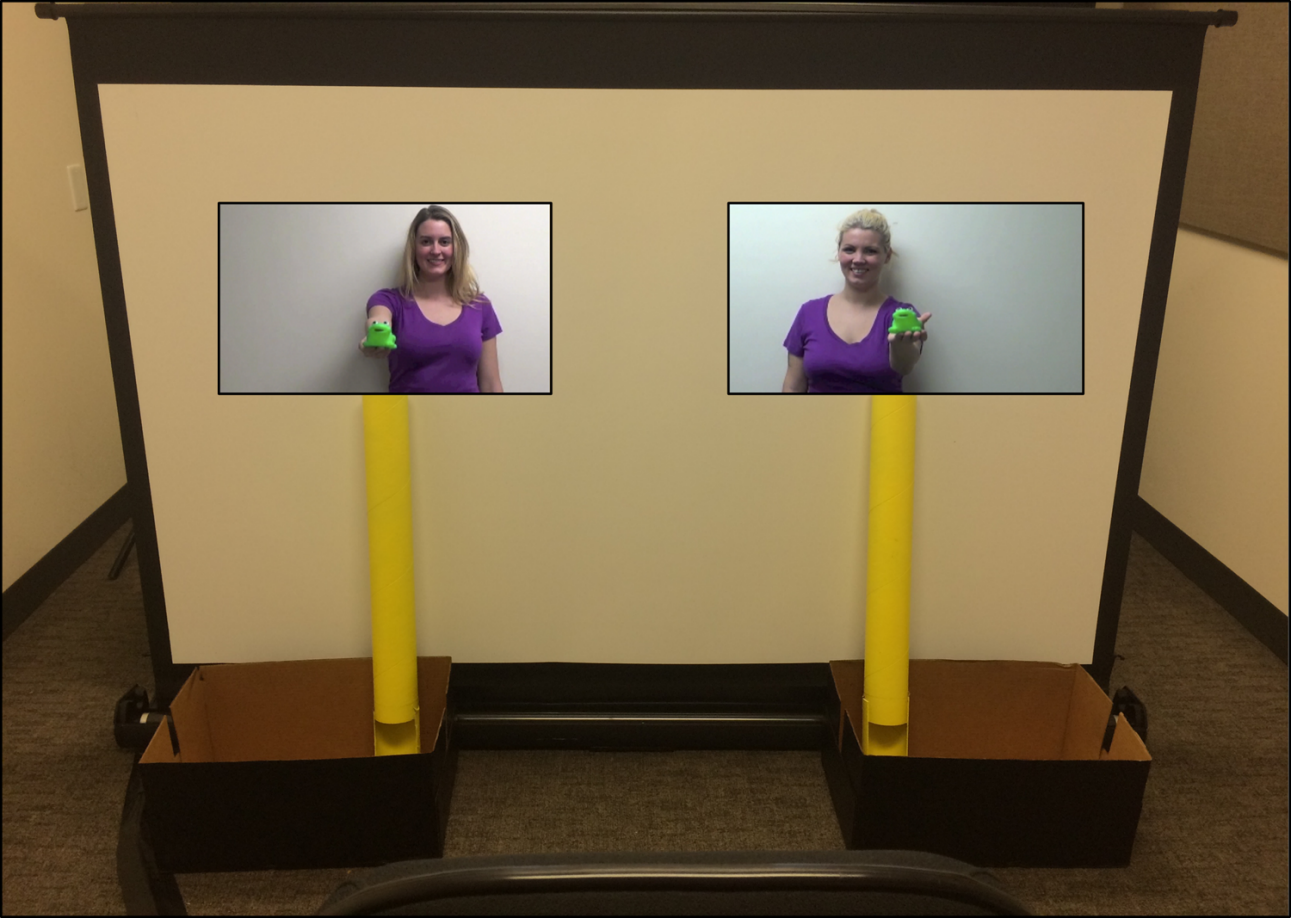
Test phase event stimuli. The distributors showed identical toys to the participant before appearing to drop the toys into the real containers underneath each video. Prior to the event, unbeknownst to the infant, the experimenter placed the toys from the video in the containers, creating the illusion that the actors on screen had dropped physical toys into the containers. Infants were then prompted to approach one of the two containers.

During the experiment, the infant sat on their parent’s lap approximately 76 centimeters in front of a projector screen. The parent was seated in a rolling chair, such that they could turn away from the screen between trials. Opaque containers were used throughout the task, consisting of a cardboard box with an attached tube. The session was recorded on video and each participant’s attention was verified by two independent coders from video after the completion of the session. The experiment consisted of practice trials, distribution events, and a test trial (see Figs 1 and 2 for visual depictions of task).

#### Practice trials

The purpose of the practice trials was to familiarize infants with the action of picking up a toy out of the container, and to increase their likelihood of completing the fairness task. In a first set of practice trials, “live practice trials”, infants were seated on their parent’s lap in front of the opaque container while an experimenter sat on the ground on the opposite side of the container, across from the infant. The experimenter showed a toy (a red fish) to the infant then dropped it into the tube of the container. After the toy fell though the tube into the bottom of the container, the parent placed the infant on the ground in front of the container, and the experimenter encouraged the infant to take the toy from the container. After the infant either retrieved the toy, or after 1 minute had elapsed and the infant did not retrieve the toy, the experimenter moved on to the next trial. Infants were given the opportunity to retrieve the toy on up to four trials; the experimenter advanced to the second set of practice trials after the infant either retrieved the toy on a total of two trials, or after four trials were administered (if the infant did not retrieve the toy on two trials).

In a second set of practice trials, “virtual practice trials”, the container was placed underneath a projector screen with a different toy (a blue octopus) already placed inside (unbeknownst to the infant). The infant watched a video in which a woman showed the toy to the infant and then appeared to drop the toy into the tube of the container. The tube of the container was aligned with the woman’s hand on screen to create the illusion that the toy fell into the tube. After watching the video, the infant was placed on the ground in front of the container and the experimenter encouraged the infant to retrieve the toy. As in the live practice trials, infants were given the opportunity to retrieve the toy on up to four trials; the experimenter advanced to the distribution event after the infant either retrieved the toy on a total of two trials, or after four trials were administered (if the infant did not retrieve the toy on two trials).

#### Distribution event

The purpose of the distribution event was to teach infants that one actor distributes crackers fairly (i.e., “fair distribution” events) and another distributes crackers unfairly (i.e., “unfair distribution” events). Parents were asked to close their eyes, in order to remain unbiased, while the infant watched the distribution event on a projector screen. Each event involved three Caucasian female actors: one distributor, that had a bowl of crackers, and two recipients, who were seated on each side of the distributor, each with an empty plate. Each distribution video began with a distributor and recipients greeting each other by stating, “Hello!” Following the greeting, the distributor held up the bowl of crackers and exclaimed, “Mmm, yummy!” Each recipient then pushed their empty plate towards the distributor while simultaneously saying, “Please”. The identity of the distributor varied across videos, such that there were two different distributors, whereas the recipients always remained the same.

In “fair distribution” videos, infants watched a fair actor distribute crackers equally between two recipients (3:3 distribution; see Fig 1A). In “unfair distribution” videos, a different unfair actor distributed more crackers to one recipient than the other recipient (5:1 distribution; see Fig 1B). After each distribution event, the distributor held up her empty bowl and exclaimed, “There, all gone!” Each video was presented twice, such that each infant saw a total of four videos (i.e., two fair distribution videos and two unfair distribution videos). Following the series of distribution events, parents were instructed to turn their chair to face the back wall so the experimenter could set up for the test trial.

#### Test trial

The test trial assessed whether infants showed a preference for the fair over the unfair individual. The test trial began with videos of both the fair and the unfair distributors appearing on either side of the screen simultaneously. In each video, the distributors showed identical toys (a bright green frog) to the participant before appearing to drop the toys into the real containers underneath each video (see Fig 2). As in the second group of practice trials, the distributors hands perfectly aligned with the tube of the container. Infants were unable to see into the container. Unbeknownst to the infant, the experimenter had already placed identical toys in the containers before each test trial, thus creating the illusion that the actors on screen had dropped physical toys into the containers. The participant was then placed on the floor in the center between the video images of the two distributors and the experimenter encouraged the infant to choose a toy. The experimenter recorded which distributor the infant took a toy from. Infants received up to three opportunities to retrieve the toy.

#### Counterbalancing

The order of the fair and unfair distribution videos (fair first versus unfair first), the identity of the fair actor, and which distributor was on the infants’ right side during the test trial were counter-balanced across participants.

### Coding and reliability

After the completion of each session, the primary experimenter coded infants’ duration of looking to the distribution videos, while blind to condition, using JHab attention coding software [34]. Another trained reliability coder, also blind to condition, independently coded distribution videos for a subset of 10 infants. Looking times between the primary coder and reliability coder were highly correlated (*r*(118) = .98, *p* < .001). Because the looking times between the primary coder and reliability coder were so highly correlated, all analyses use the primary coder’s looking times.

## Results

### Success on practice trials

To ensure that infants successfully learned how to play the game, and retrieve the toy from the containers, we first analyzed infants’ performance on the practice trials. Infants successfully retrieved the toy from the container in both “live practice” trials (*M*_13-month-olds_ = 78.57%, *SE* = 10.01%; *M*_17-month-olds_ = 93.75%, *SE* = 6.25%) and “virtual practice” trials (*M*_13-month-olds_ = 85.71%, *SE* = 22.91%; *M*_17-month-olds_ = 87.50%, *SE* = 21.88%).

### Attention to distribution videos

We examined infants’ attention to the distribution events in order to confirm that infants encoded the relevant information concerning actors’ fairness. Looking times were converted to proportion scores calculated as infants’ duration of attention to the distribution video over the entire length of the distribution video. We then calculated infants’ average attention to the distribution videos by calculating the average proportion of attention across all four videos.

Overall, infants’ attention to the distribution events was high (*M* = 98.43%, *SE* = .68%). Seventeen-month-olds’ were marginally more attentive to the distribution events (*M* = 99.81%, *SE* = .14%) than 13-month-olds (*M* = 96.85%, *SE* = 1.36%), *t*(13) = -2.16, *p* = .05. Across age groups, infants were highly attentive to both the fair (*M* = 98.40%, *SE* = .51%*)* and unfair distribution events (*M* = 97.88%, *SE* = .64%), *t*(29) = 1.55, *p* = .13. Thus, infants across both age groups successfully encoded both fair and unfair distribution events.

### Infants’ test trial choices

There were no effects of gender, side (left vs. right), or distributor actor identity (actor A vs. B) on infants’ selection during the test trial (all *p’s* > .05).

**Fig 3:**
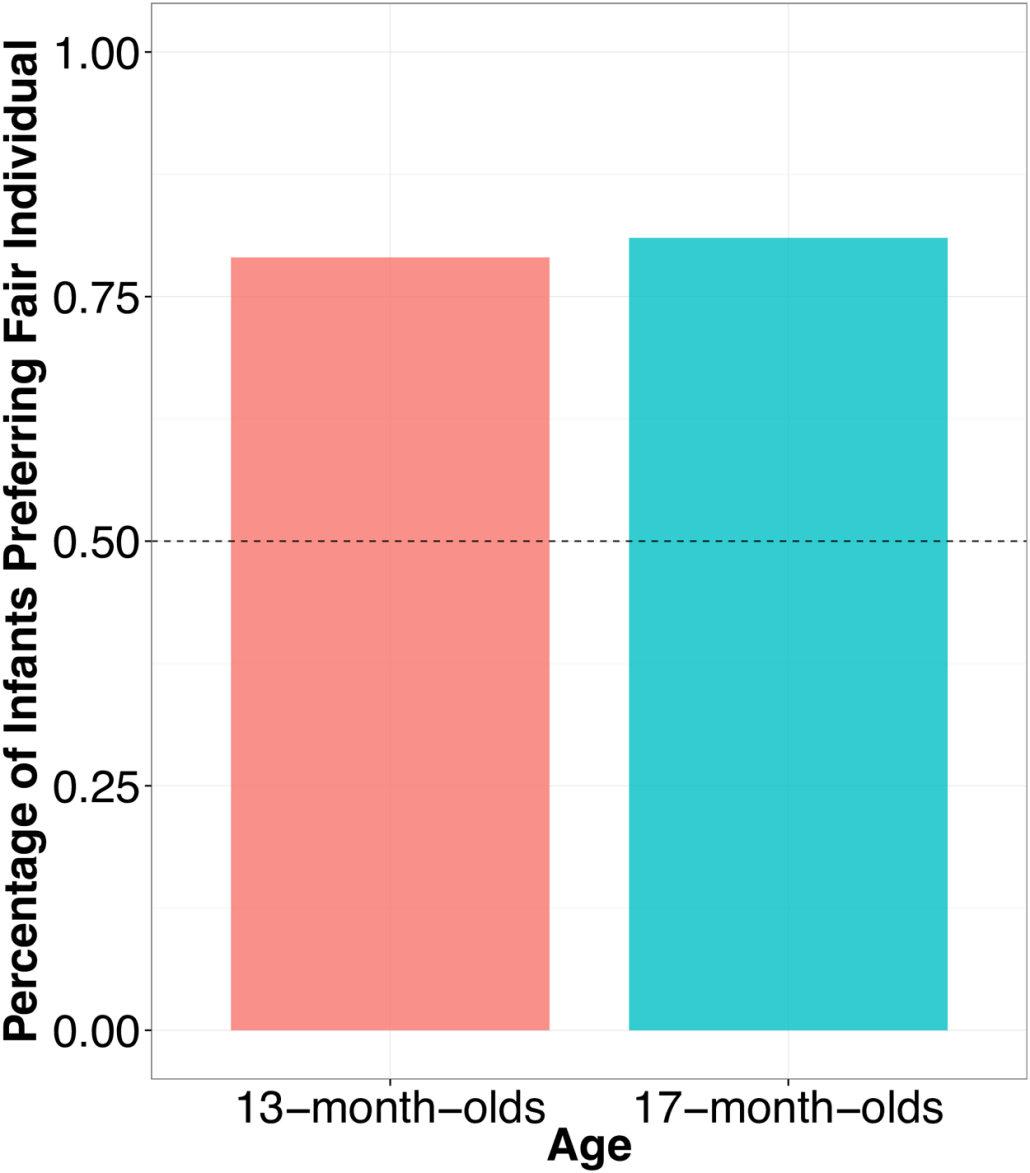
Fairness preferences. Percentage of infants who chose to interact with the fair, over the unfair, distributor. Infants across both age groups selected the fair distributor at rates above chance (i.e., .50).

The central analysis of interest was infants’ selection of the fair actor during the test trial. Because we had a directional prediction that infants would selectively choose the fair actor at rates *above* chance, one-tailed *p*-values are reported. To investigate whether infants selected the fair actor above chance on the test trial exact binomial tests were performed within each age group. Thirteen-month-olds chose the toy from the fair distributer over the unfair distributer at test significantly above chance, with 11 out of 14 infants (79% of infants) choosing the toy from the fair distributer, *p* = .03, 95% CI = [.53–1.00], Fig 3. Seventeen-month-olds chose the toy from the fair distributer over the unfair distributer at test significantly above chance, with 13 out of 16 infants (81% of infants) choosing the fair distributor, *p* = .01, 95% CI = [.58–1.00], Fig 3. Critically though, there were no significant differences found between 13- and 17-month-olds in their fair actor choice during the test trial, *χ*^*2*^(1) = 0.03, *p* = 1.0. The lack of a developmental difference suggests that infants across both age groups have a preference for interacting with fair agents over unfair agents.

One possible explanation for this pattern of results is that infants who chose the fair distributor were simply more attentive during the distribution events. This was not the case: across age groups, infants were highly attentive to the distribution events whether they chose the fair distributor (*M* = 98.65%, *SE* = .64%*)* or unfair distributor (*M* = 97.56%, *SE* = 2.44%), *t*(28) = -.671, *p* = .51.

## Discussion

The present study found that by 13 months of age, infants are able to keep track of an individual’s prior history of behaving fairly or unfairly, and use this information to guide their selection of social partners. Here, infants were exposed to two different potential social partners: one who distributed resources to third parties equally, and another who distributed resources to third parties unequally. After viewing both interactions, infants preferred to socially interact with (i.e., take a toy from) the individual who distributed resources equally. This preference was seen across development: at 17 months, infants also preferred to socially engage with individuals who had previously acted fairly towards others. Together, these findings fill in key gaps in the developmental timeline of infants’ ability to use fairness information in their consideration of potential social partners.

These findings also provide important insights into how a preference for fairness relates to the development of other markers of fairness sensitivity. During infancy, there is not only an expectation that resources should be distributed fairly, as shown in previous research [13,35], infants also assign a positive valence to those individuals that do so, and actively select those individuals as social partners. These converging findings demonstrate that even as infants’ fairness representations are first starting to develop, their understanding and use of fairness information is quite complex. These findings also demonstrate that the time between 10 and 13 months may be a critical period in the development of infants’ sensitivity to fairness: at this time multiple, potentially distinct, processes related to infants’ fairness understanding simultaneously develop.

These early emerging processes may play a defining role in the development of more complex moral intuitions, and may help explain why humans, but not other primates, eventually acquire a full-fledged sense of morality. Though nonhuman primates possess a sensitivity to fairness, they do not do so to the same extent as humans. For example, although nonhuman primates show some signs of distributive justice, and will reject unequal distributions that favor another individual [36], they will not sacrifice their own resources to equalize a distribution, a tendency that is present in humans by age 3 [37]. Thus, the early onset of a mature set of skills related to fairness sensitivity may provide a strong foundation upon which more flexible, uniquely-human moral behaviors may develop.

In addition to documenting the development of infants’ preference for fair individuals, the current study also provides insight into a novel way of assessing infants’ social preferences more broadly. Here, we designed a paradigm (adapted from [33]) that concurrently combined pre-recorded stimuli with an interactive, forced-choice task. Specifically, we created the illusion that infants had the choice to socially interact an individual, who had either previously acted fairly or unfairly, on a projector screen. In doing so, we were able to capture infants’ naturalistic social behavior while also preserving the experimental control that is inherent in using pre-recorded stimuli. Finally, in using this novel paradigm, we demonstrated that infants’ previously established fairness preferences [25] extend not only across different age groups, but also across different contexts. Traditionally, research on infants’ social preferences require infants to establish their choice by reaching. In the current study, infants had to combine walking/crawling together with reaching and searching behaviors to make their social selection. The use of a more physically and cognitively demanding measure provides important insights into the nature of infants’ fairness representations: it demonstrates that infants have a strong, deliberate, and enduring preference for fair individuals.

Infants’ systematic preference for fair individuals as social partners, across ages and contexts, highlights that infants are active agents in shaping their early social and cognitive development. When infants select a social partner, they structure the kinds of future experiences they will have, and types of information they will subsequently be exposed to. The current finding that infants are strategic in their selection of social partners is consistent with a “partner choice” model of human behavior, which posits that selective partner choice, favoring prosocial individuals, is an adaptation for uniquely-human cooperative tendencies more generally [38,39].

### Future directions

The current findings demonstrate that infants prefer to interact with individuals who behave fairly. However, they do not explain why infants have this preference. Thus, a question for future research is to investigate why infants prefer fair individuals over unfair individuals as social partners. One possibility is that unfair individuals violate infants’ expectations about how third party interactions should function, and assign a negative valence to that transgression. Whether infants understand that this norm violation is social in nature (e.g., that is not how people *typically* act) or moral (e.g., that is not how people *should* act) is an important question for future research. Research has begun to provide evidence for the latter hypothesis by demonstrating that infants assign negative valence to individuals who violate moral norms, and positive valence to those who behave consistent with moral norms [19]. To provide further evidence for this hypothesis, future research should test whether infants also assign negative valence to transgressions that are not moral in nature (e.g., violations in normative social or physical behavior). If infants uniquely assign negative valence to transgressions that are moral in nature, it will provide important evidence that infants understand that fairness violations are moral transgressions.

An additional interpretation of the current findings is that infants preferred to take an offered toy from the fair actor because they trusted that the fair actor would provide them with a toy. During the distribution event, it appeared as though both the fair and unfair actors placed toys inside boxes for the infant. However, infants could not see inside the boxes. Thus, it is possible that infants only expected the fair actor’s box to have a toy in it, which is one possible explanation for why they systematically approached and took a toy from the fair actor’s box. Future research could test this hypothesis by assessing the degree to which infants trust fair versus unfair individuals.

Another way in which future research may further inform our understanding of the nature of infants’ fairness representations is by testing whether infants understand the intentional nature of fair behaviors, and whether they use this information in their consideration of potential social partners. For instance, adults, who have a more mature representation of fairness, prefer fair individuals because of the underlying intentions that drive their behaviors, not because the results of those individuals’ behaviors happen to be fair [40]. Thus, an avenue for future research will be to investigate whether infants prefer individuals who intend to distribute resources fairly more than individuals who unintentionally (e.g., accidentally) distribute resources fairly.

In addition to more precisely documenting the nature of infants’ preference for fair individuals, future research should also investigate how this preference develops. Are there specific socialization experiences that shape infants’ preference for fair individuals, or are these preferences universal intuitions that will emerge regardless of the types of early experiences infants have? Previous research has demonstrated that other aspects of infants’ fairness understanding is shaped by early experiences. For instance, the presence of siblings is related to infants’ fairness expectations [35], and both parental empathy and previous experience sharing is correlated with early prosocial behaviors [41]. Future research should test the extent to which these variables also influence infants’ ability to use fairness information when selecting a social partner. Relatedly, cross-cultural research on infants’ preference for fair individuals can highlight the degree to which the current findings are shaped by culture-specific values.

### Conclusion

By 13 months, infants use fairness information to form evaluations of others, and use this information to guide their future behaviors, choosing to interact with fair over unfair individuals. The importance of fairness information in shaping infants’ social preferences is continuous throughout development: across the second year of life, infants continue to factor fairness information into their decisions about who to select as a social partner. These findings build on previous research by demonstrating that infants not only expect resources to be distributed fairly, they also preferentially interact with those who have previously done so. Infants’ ability to use fairness information in their consideration of social partners, from a very early age, may play a critical role in the development of more complex prosocial behaviors related to morality more broadly.
